# Urine neutrophil gelatinase-associated lipocalin is an early marker of acute kidney injury in critically ill children: a prospective cohort study

**DOI:** 10.1186/cc6089

**Published:** 2007-08-02

**Authors:** Michael Zappitelli, Kimberly K Washburn, Ayse A Arikan, Laura Loftis, Qing Ma, Prasad Devarajan, Chirag R Parikh, Stuart L Goldstein

**Affiliations:** 1 Texas Children's Hospital, Fannin Street, Houston, Texas 77030, USA; 2 Cincinnati Children's Hospital Medical Center, Burnet Avenue, Cincinnati, Ohio 45229-3039, USA; 3 Yale University School of Medicine, Campbell Avenue, West Haven, Connecticut 06516, USA

## Abstract

**Introduction:**

Serum creatinine is a late marker of acute kidney injury (AKI). Urine neutrophil gelatinase-associated lipocalin (uNGAL) is an early marker of AKI, where the timing of kidney injury is known. It is unknown whether uNGAL predicts AKI in the general critical care setting. We assessed the ability of uNGAL to predict AKI development and severity in critically ill children.

**Methods:**

This was a prospective cohort study of critically ill children. Children aged between 1 month and 21 years who were mechanically ventilated and had a bladder catheter inserted were eligible. Patients with end-stage renal disease or who had just undergone kidney transplantation were excluded. Patients were enrolled within 24 to 48 hours of initiation of mechanical ventilation. Clinical data and serum creatinine were collected daily for up to 14 days from enrollment, and urine was collected once daily for up to 4 days for uNGAL measurement. AKI was graded using pRIFLE (pediatric modified Risk, Injury, Failure, Loss, End Stage Kidney Disease) criteria. Day 0 was defined as the day on which the AKI initially occurred, and pRIFLEmax was defined as the worst pRIFLE AKI grade recorded during the study period. The χ^2 ^test was used to compare associations between categorical variables. Mann-Whitney and Kruskal-Wallis tests were used to compare continuous variables between groups. Diagnostic characteristics were evaluated by calculating sensitivity and specificity, and constructing receiver operating characteristic curves.

**Results:**

A total of 140 patients (54% boys, mean ± standard deviation Pediatric Risk of Mortality II score 15.0 ± 8.0, 23% sepsis) were included. Mean and peak uNGAL concentrations increased with worsening pRIFLEmax status (*P *< 0.05). uNGAL concentrations rose (at least sixfold higher than in controls) in AKI, 2 days before and after a 50% or greater rise in serum creatinine, without change in control uNGAL. The parameter uNGAL was a good diagnostic marker for AKI development (area under the receiver operating characteristic curve [AUC] 0.78, 95% confidence interval [CI] 0.62 to 0.95) and persistent AKI for 48 hours or longer (AUC 0.79, 95% CI 0.61 to 0.98), but not for AKI severity, when it was recorded after a rise in serum creatinine had occurred (AUC 0.63, 95% CI 0.44 to 0.82).

**Conclusion:**

We found uNGAL to be a useful early AKI marker that predicted development of severe AKI in a heterogeneous group of patients with unknown timing of kidney injury.

## Introduction

Severe acute kidney injury (AKI) increases morbidity and mortality of hospitalized patients [1-3]. Recent evidence suggests that a small reduction in renal function, indicated by serum creatinine (SCr), is an independent predictor of mortality and length of hospital stay [1,4]. Laboratory research has revealed that early intervention may be essential in preventing the pathophysiologic events that lead to AKI [5,6]. Unfortunately, SCr – the main AKI biomarker used in the clinical setting – is a late marker of reduced glomerular filtration rate, which limits ability to detect AKI early and to initiate clinical therapeutic studies. Therefore, recent research has focused on identifying earlier biomarkers of AKI [7-12].

Neutrophil gelatinase-associated lipocalin (NGAL), a ubiquitous 25 kDa protein, was isolated as a potential biomarker of AKI using genomic microarray technology [12,13]. NGAL is generally expressed in low concentrations, but it increases greatly in the presence of epithelial injury and inflammation [12,14,15]. Mishra and coworkers [16] observed a significant rise in uNGAL (uNGAL) 2 days before the rise in SCr in children with AKI following cardiopulmonary bypass (CPB). These findings have now been confirmed in a prospective study of adults who developed AKI after cardiac surgery [17], which found uNGAL to be significantly elevated by one to three hours after the operation. Other human studies [18-20] demonstrated a strong relationship between uNGAL and AKI in renal transplantation, diarrhea-associated hemolytic-uremic syndrome, and lupus nephritis.

It is unknown whether the association between uNGAL and AKI can be generalized to the critical care setting, in which the population is heterogeneous and AKI etiology and timing are often unclear. Furthermore, the prevalence of sepsis in the intensive care unit (ICU) may limit the use of uNGAL as a specific biomarker of kidney injury. We studied uNGAL concentrations in a group of critically ill children with the following goals: to determine whether there is an association between uNGAL and AKI in this heterogeneous group; to evaluate the effect of sepsis and illness severity on the use of uNGAL to predict AKI; to determine the extent to which uNGAL concentrations increase before SCr in the setting of an unknown timing of initial kidney injury; and to evaluate the sensitivity and specificity of uNGAL to predict the clinical course of AKI.

## Materials and methods

### Study design and subject selection

This study was performed concurrently with a prospective observational study that validated pRIFLE (pediatric modified Risk, Injury, Failure, Loss, End Stage Kidney Disease) criteria for defining AKI in critically ill children [21]. Patients aged 1 month to 21 years, admitted to the pediatric ICU (PICU), who received mechanical ventilation and underwent indwelling bladder catheterization, were eligible for enrollment. Patients with end-stage renal disease and who had just undergone renal transplantation were excluded. Patient care givers provided written informed consent for the child to participate in the descriptive study of AKI and for collection of urine samples. The study protocol and consent forms were approved by the Baylor College of Medicine Human Subjects Institutional Review Board before study initiation.

### Clinical data collection

The following clinical variables were evaluated: patient age, sex, height, and weight; admission and discharge diagnoses; vasopressor use (yes/no) and number of vasopressors used; renal replacement therapy provision; and 28-day mortality. Patients with an admission or discharge diagnoses of sepsis, septic shock, or systemic inflammatory response syndrome were classified as having sepsis. The Pediatric Risk of Mortality (PRISM) II score (a measure of severity of illness/mortality risk) was calculated on the day of ICU admission [22].

### Laboratory data collection

SCr values were obtained prospectively as part of routine patient care from the day of enrollment up to 14 days of the study (or until PICU discharge if this occurred before 14 days). At study completion, SCr values from PICU admission to study enrollment were recorded retrospectively. Estimated creatinine clearance (eCCl) was calculated using the Schwartz formula [23]. Patients were classified daily by pRIFLE criteria for AKI, using changes in eCCl from baseline eCCl (Table 1). Each patient's first AKI occurrence using pRIFLE criteria and the worst pRIFLE status (pRIFLEmax) attained over 14 days were recorded. Baseline renal function was defined as the lowest known SCr value during the preceding 3 months. Patients without known prior SCr were assumed to have normal baseline renal function and assigned a baseline eCCl of 120 ml/min per 1.73 m^2^. This cutoff was chosen because the Schwartz eCCl overestimates glomerular filtration rate. For those patients with no known baseline SCr and a PICU admission eCCl greater than 120 ml/min per 1.73 m^2^, their PICU admission eCCl was recorded as their baseline renal function.

**Table 1:** Pediatric modified pRIFLE criteria for AKI using changes in estimated creatinine clearance

pRIFLE stratum	Change in eCCl
Risk (R)	eCCl decrease by 25% from baseline renal function
Injury (I)	eCCl decrease by 50% from baseline renal function
Failure (F)	eCCl decrease by 75% from baseline renal function or eCCl < 35 ml/min per 1.73 m^2^

The original pediatric Risk, Injury, Failure, Loss, End Stage Kidney Disease criteria [21] for acute kidney injury (AKI) also include pRIFLE L (loss) and pRIFLE E (end stage kidney disease), identifying those patients who require dialysis for periods longer than 30 days. eCCl, estimated creatinine clearance; pRIFLE, pediatric modified Risk, Injury, Failure, Loss, End Stage Kidney Disease.

### Urine specimen collection

**Figure 1:**
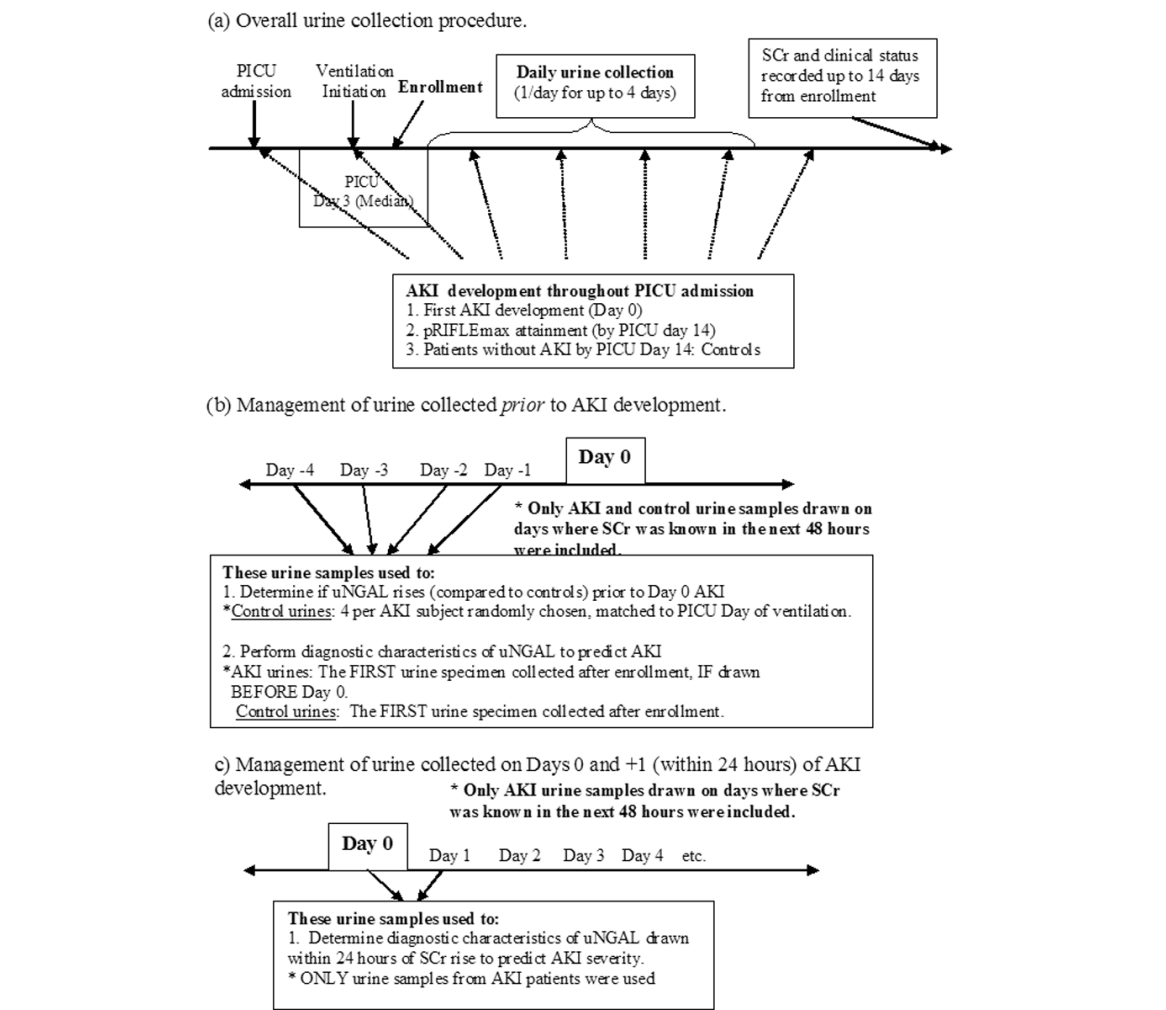
Description of urine collection procedures and use of urine specimens with reference to analytic time points. **(a) **Overall urine collection procedure. The image shows that study enrollment began shortly after initiation of ventilation and that urine was collected once per day for up to 4 days if possible. **(b) **Acute kidney injury (AKI) urine specimens collected before AKI development were used for assessment of urine neutrophil gelatinase-associated lipocalin (uNGAL) for early detection of AKI. **(c) **AKI urine specimens collected within 24 hours of AKI by pRIFLE (pediatric modified Risk, Injury, Failure, Loss, End Stage Kidney Disease) criteria were used to evaluate uNGAL as a marker of severity of renal injury. day 0, the first day the patient attained AKI; PICU, pediatric intensive care unit; pRIFLEmax, the worst pRIFLE stratum attained; SCr, serum creatinine; uNGAL, urine neutrophil gelatinase-associated lipocalin.

Urine specimens were collected at 14:00 hours each day, for up to four consecutive days, beginning on the day of enrollment or the following day if consent was obtained after 14:00 hours (Figure 1a). Reasons for not collecting urine samples on all four days included bladder catheterization discontinuation, hospital discharge, death, and anuria. Urine bags were emptied at 13:00 hours to allow collection of fresh urine in the following hour. Anuria was defined as less than 5 ml in the urine collection bag from the hour before collection, because this was the minimum amount required for processing and storage.

Urine processing was similar to that in previous studies [18,19], in order to limit variations in findings resulting from differences in sample handling. Urine specimens were kept on ice until they were centrifuged at 3,000 rpm at 4°C for 5 min. The supernatant was aliquoted equally into cryovials and stored at -80°C. Pre-laboratory analysis sample handling required minimal time and effort (approximately 10 min). De-Samples were shipped to Cincinnati Children's Hospital Medical Center for uNGAL and creatinine measurement; lab personnel were blinded as to any patient information and pRIFLE status. Urine samples were analyzed for NGAL using an established and validated enzyme-linked immunosorbent assay [18,19,24]. Microtiter plates were coated overnight at 4°C with a mouse monoclonal antibody directed against human NGAL (#HYB211-05; AntibodyShop, Gentofte, Denmark). All subsequent steps were performed at room temperature. Plates were blocked with buffer containing 1% bovine serum albumin, coated with 100 μl sample (urine or serum) or standards (NGAL concentrations ranging from 1 to 1000 ng/ml), and incubated with a biotinylated monoclonal antibody directed against human NGAL (#HYB211-01B; AntibodyShop) followed by avidin-conjugated horseradish peroxidase (Dako, Glostrup, Denmark). TMB substrate (BD Biosciences, San Jose, CA, USA) was added for color development, which was read after 30 min at 450 nm with a microplate reader (Benchmark Plus; BioRad, Hercules, CA, USA). Urine creatinine was measured using a quantitative colorimetric assay (Sigma Chemical Co., St. Luois, MO, USA). All measurements were taken in triplicate. The Cincinnati Children's Hospital Medical Center laboratory was blinded to the AKI status of each patient. Final uNGAL values were expressed in nanograms per milliliter and nanograms per milligram of creatinine.

### Secondary exclusion of patients and urine samples

Before statistical analysis of urine samples, patients were further excluded from this study if fewer than two SCr values were available for the duration of the admission (and not before early death) or if no urine specimens were collected throughout the study period. If patients had even one urine specimen collected, they were included.

### Data management, interpretation, and analysis

Using all urine specimens available from all patients, the mean and peak uNGAL concentrations from each patient were tabulated. Mean and peak uNGAL were compared between control individuals and those with AKI (based on the R, I, and F components of pRIFLEmax) during admission. The data were examined for an association between mean or peak uNGAL and the presence of sepsis, PRISM II scores, and mortality.

For all subsequent analyses, only data from urine samples for which SCr was known in the 48 hours after urine collection were used. We first examined whether uNGAL rises before clinical evidence of AKI becomes apparent, as determined by pRIFLE criteria. The data were arranged to define 'day 0' as the first day on which a patient sustained AKI. Urine samples collected between 72 hours before day 0 (days -3, -2, and -1; Figure 1b) and 48 hours after day 0 (days 0, +1, and +2) were compared with control uNGAL concentrations. Up to four control urine specimens per AKI urine specimen, drawn during the same day of mechanical ventilation as the AKI patient, were randomly selected for comparison using a random number generator. Some control urine specimens are represented more than once for comparison with different AKI urine specimens.

The diagnostic characteristics of uNGAL in predicting AKI were examined. The first urine specimen collected from AKI patients who had urine collected before AKI development and the first urine specimen collected from control individuals (Figure 1b) were used to calculate the sensitivity and specificity of uNGAL in predicting the onset of AKI during the next 48 hours and the onset of 'persistent' AKI durinng the next 48 hours. 'Persistent AKI' was defined as lack of complete resolution of AKI within 48 hours, as a surrogate marker of patients who had fluid responsive AKI. We only used the first urine specimen collected from these patients to simulate the collection of urine for NGAL measurement shortly after becoming 'at risk' (the day of initiation of mechanical ventilation) but before the development of AKI.

Several patients had their first urine sample collected on the day of or one day after developing AKI (within 24 hours of the first detected SCr increase, as shown in Figure 1c). We therefore evaluated the utility of uNGAL from day 0 or day +1 to predict persistent AKI and progression of AKI to a higher pRIFLEmax stratum in patients who initially satisfied the R criterion of pRIFLE.

### Statistical analysis

Urine NGAL was non-normally distributed, and therefore nonparametric testing was used to compare uNGAL concentrations between groups (Mann-Whitney test for two groups and Kruskal-Wallis test for multiple groups). Categorical variables were analyzed using the χ^2 ^test, and proportions were compared using the z-test. Diagnostic characteristics were calculated using standard 2 × 2 tables, and receiver operating characteristic curves were constructed. Analyses were performed using the Intercooled STATA^® ^statistical software package (Stata Corp., College Station, TX, USA). Values which followed a normal distribution are expressed as mean ± standard deviation and those which followed a non-normal distribution are expressed as median [interquartile range].

## Results

### Patient demographics

A total of 150 patients were enrolled in the AKI study conducted to validate the pRIFLE criteria [21]. Ten patients were excluded from urinary biomarker studies: five were anuric and for five fewer than two SCr measurements were available. The mean age was 6.3 ± 6.4 years (median 3.5 years, range 1 month to 21 years) and mean weight was 24.9 ± 21.5 kg (median 15.6 kg) for the remaining 140 subjects (75 boys [54%] and 65 girls [46%]). Nine patients had a baseline eCCl below 90 ml/min per 1.73 m^2^; three patients had an eCCl below 60 ml/min per 1.73 m^2^. The mean PRISM II score was 15.0 ± 8.0 (median 15). Thirty-two (23%) patients had a diagnosis of sepsis and 74 (53%) received vasopressors.

Mean PICU day of enrollment was 3.0 ± 1.5 days (median 3 days, range 1 to 9 days). Eighty-nine per cent of patients were enrolled on or before PICU day 4. Urine collection began on PICU day 3.0 ± 1.4 (median day 3) and day of ventilation 2.3 ± 0.9 (median day 2).

Thirty-four (24.3%) patients never sustained AKI and served as control individuals. A total of 106 (75.7%) patients developed AKI (35.7% [*n *= 50] satisfied the R criterion in their pRIFLEmax, 22.1% [*n *= 31] satisfied the I criterion in their pRIFLEmax, and 17.9% [*n *= 25] satisfied the F criterion in their pRIFLEmax). Baseline eCCl was similar between control and AKI patients (median [interquartile range] 119 [38] ml/min per 1.73 m^2 ^and 129 [87] ml/min per 1.73 m^2^, respectively; *P *> 0.05). For 82% of patients with AKI, urine collections were available between 72 hrs before and after day 0 of AKI.

Table 2 shows the characteristics of patients in the control group and for those in each pRIFLEmax stratum (namely, those satisfying the R, I, and F criteria in the pRIFLEmax for AKI). Patients in the control group were older than those in the pRIFLEmax R and I groups. PRISM II scores increased progressively with worsening pRIFLEmax strata (*P *< 0.05, Kruskal-Wallis test), and the combined mortality of patients with pRIFLEmax I and F (*n *= 56) was higher than the combined mortality of control and pRIFLEmax R patients (*n *= 84; *P *< 0.05, z-test).

**Table 2:** Patient characteristics by pRIFLEmax AKI status

Characteristic	Control (*n *= 34)	pRIFLEmax R (*n *= 50)	pRIFLEmax I (*n *= 31)	pRIFLEmax F (*n *= 25)
Age (years)	8.5 ± 6.2^a^/8 (11.0)	5.9 ± 6.7/2 (12.4)	4.4 ± 5.7/1 (8.7)	6.6 ± 6.4/4 (11.2)
PRISM II score^b^	12.5 ± 7.7/12.5 (10)	14.2 ± 7.9/15 (13)	15.9 ± 7.3/16 (9)	19.0 ± 8.0/19 (12)
Day of admission enrolled (days)	2.8 ± 1.0/3 (1)	2.8 ± 1.1/2.5 (1)	3.3 ± 2.1/3 (2)	3.1 ± 1.7/3 (2)
Day of admission of pRIFLEmax	NA	3.6 ± 3.6/2 (4)	2.5 ± 2.6/1 (2)	3.5 ± 4.0/1 (3)
Day of ventilation first urine collection	2.2 ± 0.7/2 (0.5)	2.5 ± 1.1/2 (1)	2.2 ± 0.8/2 (1)	2.3 ± 0.9/2 (1)
Days from day 0 of first urine collection^c^	NA	-0.8 ± 3.7/0 (4)	1.8 ± 2.2/1 (1)	1.7 ± 1.0/2 (1)
Male	18 (52.9)	27 (54.0)	18 (58.1)	12 (48.0)
Sepsis	7 (20.6)	4 (8.0)^d^	12 (38.7)	9 (36.0)
Dialysis	0	0	2 (6.5)	5 (20.0)
30-day mortality	3 (8.8)	4 (8.0)	6 (19.4)	7 (28.0)

Values are expressed as mean ± standard deviation/median (interquartile range) or as *n *(%). ^a^Control patients were older than those with pRIFLEmax R and I acute kidney injury (AKI; *P *< 0.05, Mann-Whitney test). ^b^Pediatric Risk of Mortality (PRISM) II score increased progressively with increasing pRIFLEmax strata (*P *< 0.05, Kruskal-Wallis test). ^c^Number of days from the day of AKI attainment that the first of four urine samples was collected for each patient. ^d^Patients in the pRIFLEmax R group had a lower proportion of sepsis, as compared with those in the pRIFLEmax I and F groups (both *P *< 0.05, z-test). pRIFLEmax, the worst pRIFLE stratum attained; pRIFLE, pediatric modified Risk, Injury, Failure, Loss, End Stage Kidney Disease.

### Association of mean and peak uNGAL concentrations with pRIFLEmax

**Figure 2:**
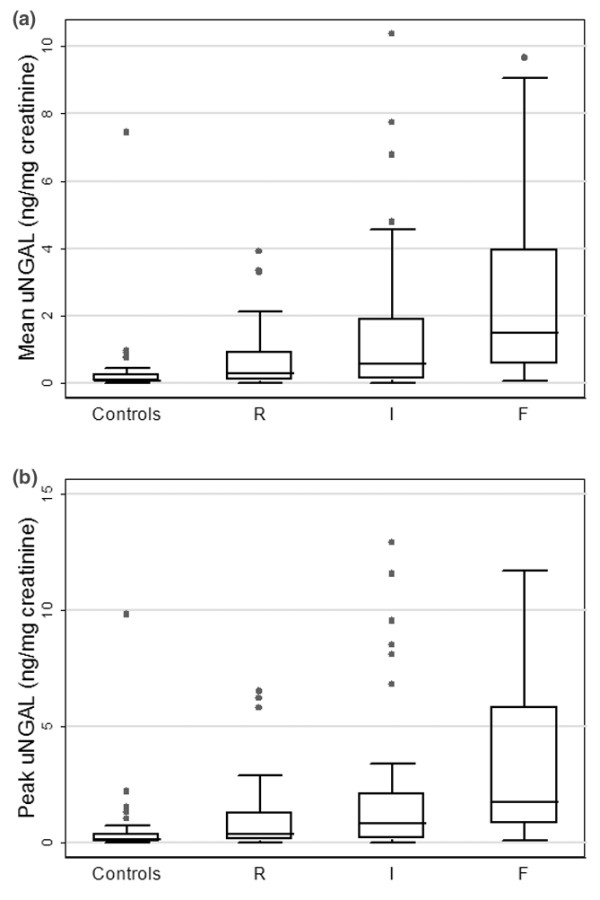
Mean and peak uNGAL concentrations. Shown are box plots of **(a) **mean and **(b) **peak urine neutrophil gelatinase-associated lipocalin (uNGAL) concentrations by pRIFLEmax strata. The mean uNGAL is the mean of uNGAL in each patient's four urine specimens, and peak uNGAL is the highest uNGAL level from each patient. AKI, acute kidney injury; pRIFLE, pediatric modified Risk, Injury, Failure, Loss, End Stage Kidney Disease; pRIFLEmax, the worst pRIFLE stratum attained; R, pRIFLEmax R AKI; I, pRIFLEmax I AKI; F, pRIFLEmax F AKI.

All urine specimens were used to calculate mean and peak uNGAL. A total of 334 urine specimens were obtained from 106 patients with AKI (3.2 specimens/patient) and 104 urine specimens were obtained from 34 controls (3.1 specimens/patient). For 75 patients urine specimens were available on all four days, for 28 patients on three days, and for 17 patients on two days; for 20 patients one urine specimen was available. Table 3 shows the mean and peak uNGAL concentrations by pRIFLEmax strata and Figure 2 illustrates the data graphically. There was a statistically significant association between worsening pRIFLEmax status and increasing mean and peak uNGAL concentrations (all *P *≤ 0.0002, Kruskal-Wallis test), whether uNGAL was expressed as nanograms per milligram creatinine or as nanograms per milliliter (Table 3). uNGAL results are subsequently presented only in nanograms per milligram creatinine, because all associations observed held true from uNGAL expressed in nanograms per milliliter, as found in previous studies [16,18].

**Table 3:** Peak and Mean uNGAL concentrations by pRIFLEmax status

Measurement	Control	pRIFLEmax R	pRIFLEmax I	pRIFLEmax F
Mean uNGAL^a^				
ng/mg creatinine	0.5 ± 1.5/0.1 (0.2)	0.6 ± 0.9^b^/0.3 (0.9)	1.7 ± 2.6^b^/0.7 (1.8)	2.8 ± 3.0^b,c^/1.5 (4.2)
ng/ml	14.2 ± 27.2/5.3 (13.2)	20.9 ± 28.1/11.6 (27.5)	58.9 ± 86.6^b^/20 (71.4)	82.7 ± 92.5^b,c^/35.0 (76.3)
Peak uNGAL^a^				
ng/mg creatinine	0.8 ± 2.0/0.2 (0.4)	1.0 ± 1.5^b^/0.4 (1.2)	2.5 ± 3.8^b^/0.9 (1.9)	3.8± 3.8^b,c^/1.8 (5)
ng/ml	24.6 ± 45.5/7.9 (20.0)	34.5 ± 47.4/14.7 (40.5)	82.9 ± 122.9^b^/25.0 (70.0)	103.2 ± 107.3^b,c^/55.0 (105.0)

Values are expressed as mean ± standard deviation/median (interquartile range). ^a^Mean and peak urine neutrophil gelatinase-associated lipocalin (uNGAL) concentrations increased with worsening pRIFLEmax acute kidney injury (AKI; all *P *< 0.0002, Kruskal-Wallis test), whether expressed in ng/mg creatinine or ng/ml. These relationships were also statistically significant when examined by one-way analysis of variance (*P *< 0.0001). ^b^Mean and peak uNGAL expressed in ng/mg creatinine was higher in patients with pRIFLEmax R, I and F AKI than in control patients (all *P *< 0.05, Mann-Whitney test); mean and peak uNGAL expressed in ng/ml was higher in patients with pRIFLEmax R, I, and F AKI than in control patients (all *P *< 0.05, Mann-Whitney test). ^c^Mean and peak uNGAL was higher in patients with pRIFLEmax F AKI than in those with pRIFLEmax R and I AKI (all *P *< 0.05, Mann-Whitney test), whether expressed in ng/mg creatinine or in ng/ml. pRIFLEmax, the worst pRIFLE stratum attained; pRIFLE, pediatric modified Risk, Injury, Failure, Loss, End Stage Kidney Disease.

### uNGAL as an early predictor of AKI

**Figure 3:**
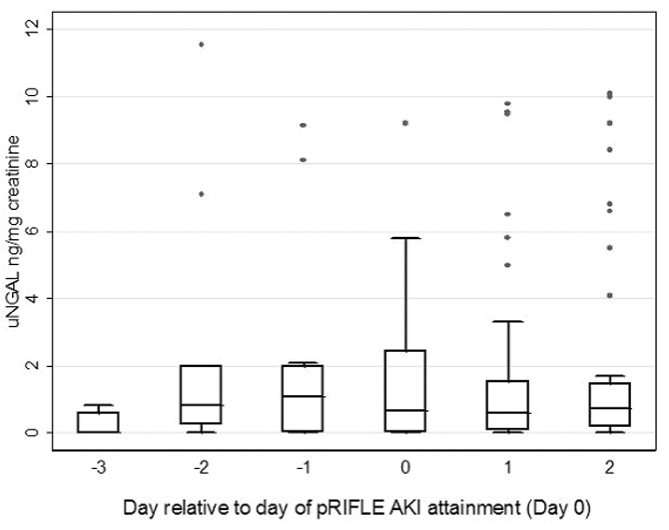
uNGAL concentrations from 3 days before to 2 days after sustaining AKI. The center lines represent the median values and the two outer lines represent the interquartile range. AKI, acute kidney injury; pRIFLE, pediatric modified Risk, Injury, Failure, Loss, End Stage Kidney Disease; uNGAL, urine neutrophil gelatinase-associated lipocalin.

Figure 3 shows the uNGAL concentrations for patients with AKI from days -3 to +2 of AKI. On day -3, uNGAL concentrations were not different from control uNGAL concentrations (median [interquartile range] 0.0 [0.6] versus 0.1 [0.2] ng/mg creatinine; *P *> 0.05, Mann-Whitney test). Whereas subsequent control uNGAL values remained low (median ranging from 0.02 to 0.1 ng/mg creatinine), AKI uNGAL concentrations were several fold higher than control uNGAL concentrations from days -2 to +2 (median [interquartile range] = 0.8 [2.0], 1.1 [2.0], 0.7 [2.0], 0.6 [1], and 0.8 [1] ng/mg creatinine on days -2, -1, 0, +1, and +2, respectively; all *P *< 0.05 versus control).

### Diagnostic characteristics of uNGAL in predicting AKI

**Figure 4:**
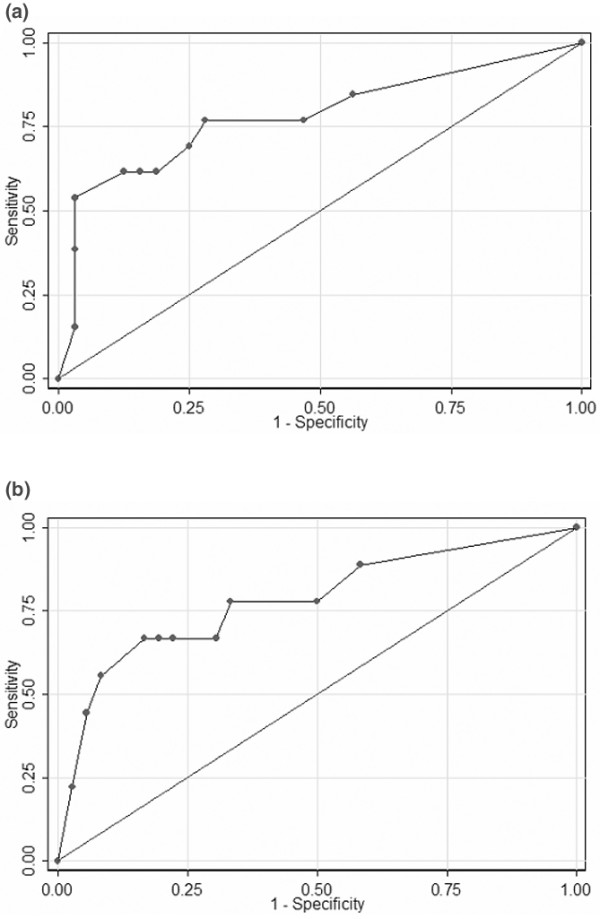
Receiver operating characteristic curve for uNGAL. Shown are receiver operating characteristic curve for uNGAL on days -2 or -1 used to predict development of **(a) **acute kidney injury (AKI) within 48 hours (area under the receiver operating characteristic curve [AUC] 0.78) and **(b) **persistent AKI within 48 hours of first urine collection (AUC 0.80).

Patients for whom the first of four urine samples was collected anytime before development of AKI (*n *= 21 urine specimens with known SCr during the 48 hours following urine collection) were analyzed to examine the diagnostic performance of uNGAL for predicting the following outcomes: development of any AKI in the next 48 hours and development of persistent AKI in the next 48 hours. The first of four urine specimens (for which SCr was known during the 48 hours following urine collection) collected from patients in the control group were also included (*n *= 24; Figure 1b). The area under the curve (AUC) for receiver operating characteristic (ROC) for uNGAL for prediction of any AKI within 48 hours of the first urine collection was 0.78 (95% confidence interval [CI] 0.62 to 0.95; Figure 4a). The AUC for diagnosing persistent AKI in the next 48 hours was 0.79 (95% CI 0.61 to 0.98; Figure 4b). The sensitivities and specificities for different uNGAL cutoffs are shown in Table 4. At the lowest evaluated uNGAL concentration cutoff of 0.05 ng/mg creatinine, sensitivity and specificity for detecting AKI in the next 48 hours were 85% and 44%; at the highest cutoff of 1.5 ng/mg creatinine, specificity was 97% with a sensitivity was 54%.

**Table 4:** Diagnostic performance of different uNGAL thresholds to detect the development of AKI and persistent AKI within 48 hours

uNGAL cutoff (ng/mg creatinine)	Sensitivity (%)		Specificity (%)		Correctly classified (%)	
	
	AKI	Persistent AKI	AKI	Persistent AKI	AKI	Persistent AKI
0.05	85	89	44	42	56	51
0.1	77	78	53	50	60	56
0.2	77	78	72	67	73	69
0.3	69	67	75	69	73	69
0.4	62	67	81	78	76	76
0.8	62	67	84	81	78	78
1	62	67	88	83	80	80
1.5	54	56	97	92	73	84

AKI, acute kidney injury; uNGAL, urine neutrophil gelatinase-associated lipocalin.

### Diagnostic characteristics of uNGAL in predicting the course of AKI

We studied urine specimens collected within two days of initiation of mechanical ventilation in AKI patients for whom the first urine sample was collected on day 0 or day 1. We examined the diagnostic ability of uNGAL to predict persistent AKI and progression of initial pRIFLE R AKI on day 0 to a worse final pRIFLEmax. The AUC for day 0/+1 uNGAL for predicting persistent AKI was 0.63 (95% CI 0.44 to 0.82), and the AUC of uNGAL for predicting worsening from pRIFLE R to pRIFLEmax I/F AKI was 0.61 (95% CI 0.32 to 0.89).

### Association of uNGAL with PRISM II, mortality, and sepsis

We observed a weak correlation between PRISM II scores and mean and peak uNGAL concentrations in patients with AKI (Spearman rho = 0.18 for both, *P *< 0.05) but not for patients in the control group (Spearman rho = -0.01 and 0.04, respectively; *P *> 0.05). There was no difference in peak or mean uNGAL concentrations between survivors and nonsurvivors when pRIFLEmax strata were examined separately (Table 5) or when the group was examined as a whole (*P *> 0.05, Mann-Whitney test).

**Table 5:** Peak and mean uNGAL concentrations in survivors and nonsurvivors, by pRIFLEmax AKI strata

Measurement (ng/mg creatinine)	Group (numbers of patients: survivors/nonsurvivors)			
	
	Control (31/3)	pRIFLEmax R (46/4)	pRIFLEmax I (25/6)	pRIFLEmax F (18/7)
Peak uNGAL^a^				
Survivors	0.6 ± 1.8/0.1 (0.4)	0.9 ± 1.4/0.4 (1.2)	2.4 ± 3.5/0.8 (2.0)	4.1 ± 3.7/2.7 (5.5)
Nonsurvivors	0.4 ± 0.3/0.2 (0.5)	1.8 ± 2.7/0.7 (3.2)	2.7 ± 5.0/0.9 (1.0)	2.8 ± 4.0/1.5 (1.8)
Mean uNGAL^a,b^				
Survivors	0.4 ± 1.3/0.1 (0.2)	0.6 ± 0.8/0.3 (0.7)	1.6 ± 2.5/0.5 (1.8)	3.1 ± 3.1/2.2 (4.2)
Nonsurvivors	0.3± 0.2/0.2 (0.4)	1.1 ± 1.5/0.6 (2.1)	1.8 ± 3.0/1.0 (1.1)	1.9 ± 2.6/1.1 (1.1)

Values are expressed as mean ± standard deviation/median (interquartile range). ^a^Peak and mean urine neutrophil gelatinase-associated lipocalin (uNGAL) concentrations were not statistically significantly different between survivors and nonsurvivors in any of the pRIFLE strata (*P *> 0.05, Mann-Whitney test). ^b^Mean uNGAL refers to the mean of all 4 urine specimens collected for each patient. pRIFLEmax, the worst pRIFLE stratum attained; pRIFLE, pediatric modified Risk, Injury, Failure, Loss, End Stage Kidney Disease.

**Figure 5:**
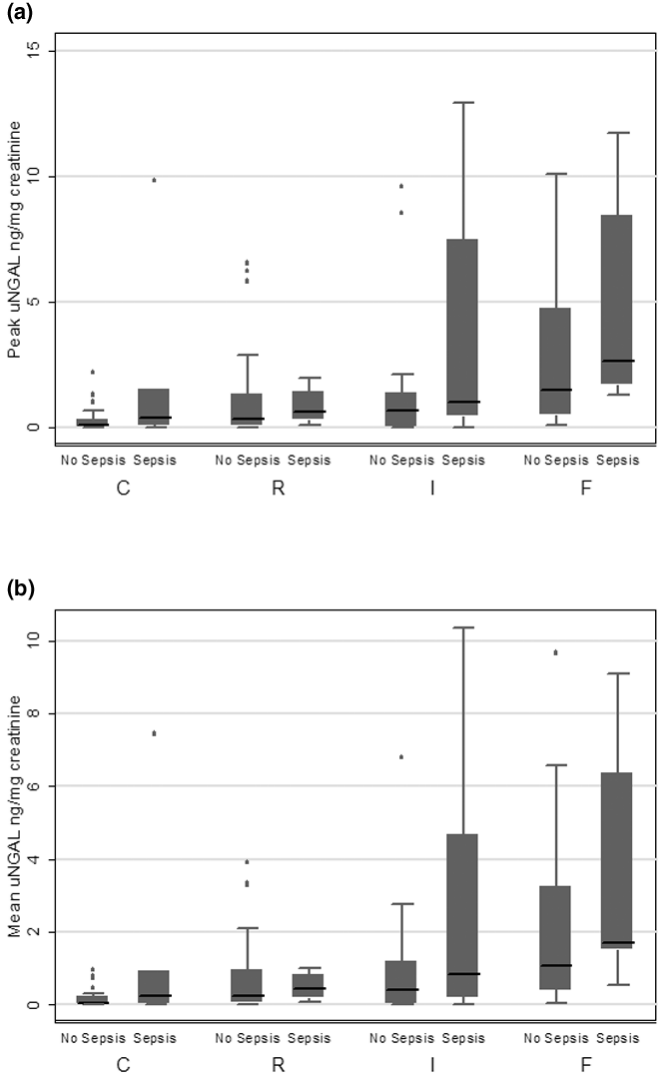
Mean and peak uNGAL concentrations according to presence or absence of sepsis. Shown are box plots of **(a) **peak urine neutrophil gelatinase-associated lipocalin (uNGAL) concentrations in patients with and without sepsis, by pRIFLEmax strata, and **(b) **mean uNGAL concentrations in patients with and without sepsis. pRIFLE, pediatric modified Risk, Injury, Failure, Loss, End Stage Kidney Disease; pRIFLEmax, the worst pRIFLE stratum attained; uNGAL, urine neutrophil gelatinase-associated lipocalin.

Thirty-two patients had a diagnosis of sepsis. One patient with a positive urine culture in the setting of a multiorganism blood infection attained pRIFLEmax R AKI with mean and peak uNGAL concentrations similar to those of other patients with pRIFLEmax R AKI, (0.6 and 0.8 ng/mg creatinine, respectively). Sixteen (50%) patients had a positive blood culture and had mean and peak uNGAL concentrations similar to those of patients diagnosed with sepsis with a negative blood culture (*P *> 0.5; data not shown). Septic patients with pRIFLEmax I/F had higher mean and peak uNGAL concentrations than did patients without sepsis (*P *< 0.05). This association was not observed in control or pRIFLEmax R patients. The relationship of increasing uNGAL values with worsening pRIFLEmax status was present in patients with and in those without sepsis (both *P *< 0.05, Kruskal-Wallis test; Figure 5), similar to when the whole group was examined (Figure 2).

## Discussion

We assessed the ability of uNGAL to predict AKI development and characterize the degree of AKI in critically ill pediatric patients. Our study is among the first to examine a urinary biomarker in a heterogeneous population in which the timing of renal insult is largely unknown [25]. Previous uNGAL studies [16,17,19,20] focused on a single renal disease entity or were conducted in patient populations in which the timing of renal insult was known or AKI development was predictable.

We found that uNGAL concentrations in AKI patients exhibited a sixfold increase in concentration that persisted from 48 hours before to 48 hours after development of AKI. The timing of uNGAL increase substantiates the findings of Mishra and coworkers [16] in their study of NGAL in children who had undergone CPB. Urinary NGAL concentrations of AKI patients in our PICU population differed from those of other groups described in the literature. For instance, uNGAL concentrations for AKI patients in our study were 200-fold to 1000-fold lower than renal transplant recipients with delayed graft function [19] or with diarrhea-positive hemolytic-uremic syndrome who required dialysis [20], and were 5-fold to 15-fold higher than observed in the pediatric CPB cohort [16]. These differences in uNGAL concentration are expected because kidney injury associated with primary renal insults may be more severe than that in most patients included in our study, but our patients were probably more severely ill, with a higher proportion having sepsis, than children undergoing CPB. This finding also confirms the need for future research to evaluate uNGAL in different renal disease subgroups, in order to understand fully how best to use uNGAL to diagnose AKI. Future research should evaluate how specific diagnoses and medications affect uNGAL levels, independently of AKI and sepsis.

We also found mean and peak uNGAL concentrations to be associated with increasing pRIFLEmax strata, and uNGAL concentrations from 24 and 48 hours before AKI development predicted which patients would develop persistent AKI, with good AUCs in the range of 0.78 to 0.79. Although the AUCs in our study were not as robust as in previous studies, as noted above, our study differed in the following ways: AKI timing in our patients was unknown (unlike the CPB and immediate post-renal transplant patient populations, where NGAL concentrations can be tested at different specific time points after the event that incites AKI); and our population was heterogeneous as compared with uNGAL studies in primary renal disease. Given these circumstances, we suggest that the AUCs generated from our data indicate that uNGAL performed reasonably well in terms of predicting AKI occurrence and severity before AKI development. The diagnostic characteristics of uNGAL in detecting AKI within 48 hours (Table 4) suggest that a uNGAL cutoff value of 0.2 to 0.3 ng/mg creatinine provides the maximum sensitivity for a given specificity in this patient population. Further studies in other critically ill populations should be performed to confirm the validity and generalizability of these cutoff values.

Previous reports have suggested that another urinary AKI biomarker, interleukin-18, is elevated in critically ill adult patients who later die, which can complicate the interpretation of urinary interleukinn-18 in the most severely ill patients [25]. Although we observed a weak correlation between uNGAL and PRISM II scores for the entire cohort, uNGAL concentrations were no different between survivors and nonsurvivors in control patients or at each pRIFLE stratum. Absence of confounding by severity of illness is a desirable quality in an AKI biomarker, because elevated levels are unlikely to be due solely to illness severity or impending death. In addition, despite the known association between uNGAL and inflammation [14,15], we observed an association between pRIFLEmax strata and increasing uNGAL concentrations in patients with sepsis (whose level of systemic inflammation is probably much higher than the intrarenal inflammation associated with AKI) and in those without sepsis, suggesting that uNGAL is an independent AKI biomarker.

Our study had several limitations. Because we studied only the most critically ill patients (those who required mechanical ventilation), many patients had already developed AKI at the time of study enrollment. As a result, we obtained urine from only a subgroup of patients for uNGAL assessment before AKI development. We also only assessed uNGAL and SCr once daily, and therefore we could have missed earlier rises in both markers. We excluded patients whose urine output was less than 5 ml during the hour before urine sampling. Such a strategy could have led to potential exclusion of many patients who were not truly anuric, but we only excluded five patients because of low urine output in that hour. Although we previously validated the pRIFLE criteria, which are based on changes in eCCl [21], use of eCCl to define AKI must be interpreted with caution because eCCl formulae were originally derived in stable patients who were not critically ill. The main reservation associated with use eCCl is related to variability in SCr concentrations in the non-steady state. Therefore, future research must attempt to identify other serum markers of glomerular filtration rate, such as cystatin C, which may not be greatly affected by rapid alterations in steady state serum levels and may provide a more accurate 'gold standard' against which early AKI biomarkers can be tested. Finally, it is possible that the characteristics of uNGAL may not be the same in clinical settings that we did not specifically assess (for example, AKI due to nephrotoxic medication versus fluid-related acute tubular necrosis). Our sample size was not large enough to perform multiple subgroup analyses, and we chose to focus on septic as opposed to nonseptic patients. Future research must elucidate the utility of uNGAL as a diagnostic marker of AKI in specific AKI etiologic entities.

Although our cohort represents a relatively large pediatric AKI cohort, subgroup analyses must be viewed with caution. Although we studied only the most critically ill patients, we observed a 14.2% mortality rate. A larger sample size would be required to provide adequate power for assessment of a weak association between uNGAL and mortality. uNGAL concentrations were neither sensitive nor specific for predicting the course of AKI once SCr was already elevated. Although this finding suggests that uNGAL is not a good predictor of AKI course once AKI has developed, our sample size might have been too small to substantiate firmly this negative finding. Other urinary markers should be examined for their utility to determine AKI severity once SCr is already elevated, given that SCr is still the standard for diagnosing AKI. Finally, it may not be appropriate to extrapolate the results we obtained to adult populations, who may exhibit greater degrees of chronic inflammation.

## Conclusion

AKI has emerged as an important health problem in hospital patients. Recent efforts to define and characterize AKI [26,27] have led to studies of early AKI detection and will ultimately contribute to improvements in AKI outcomes. Data from the present study suggest that uNGAL serves well in predicting AKI before a rise in SCr becomes apparent and who will have persistent AKI. It is likely that no urinary biomarker will be able to perform all tasks of predicting AKI, for instance determining both severity and duration, as well as portending recovery. Although the use of urinary biomarkers is currently limited to research investigations, and sample processing can only occur at a few laboratories, the ultimate goal will be to develop a biomarker panel in a urine dipstick format that permits rapid assessment of biomarker threshold concentrations.

## Key messages

• uNGAL concentrations rose 48 hours before a 50% or greater rise in SCr, in a heterogeneous group of critically ill children.

• uNGAL is good diagnostic marker of AKI in settings in which the timing of kidney injury is unknown.

• Children with sepsis have higher uNGAL concentrations than do those without sepsis, but the relationship between uNGAL and AKI is maintained.

• uNGAL may not be a good predictor of AKI severity, once SCr rise has already occurred.

## Abbreviations

AKI = acute kidney injury; AUC = area under the receiver operating characteristic curve; CI = confidence interval; CPB = cardiopulmonary bypass; eCCL = estimated creatinine clearance; PICU = pediatric intensive care unit; pRIFLE = pediatric modified Risk, Injury, Failure, Loss, End Stage Kidney Disease criteria; PRISM = Pediatric Risk of Mortality; SCr = serum creatinine; uNGAL = urine neutrophil gelatinase-associated lipocalin.

## Competing interests

PD has entered into a licensing agreement for the NGAL assay with Biosite Inc. (plasma NGAL) and Abbott Diagnostics (uNGAL).

## Authors' contributions

MZ participated in urine processing, performed the statistical analysis, and drafted the manuscript. KKW participated in the project coordination, recruitment, urine collection/processing, and drafting of the manuscript. AAA participated in the project coordination, recruitment, urine collection/processing, data interpretation, and drafting of the manuscript. LL participated in the study design and data interpretation. Qing Ma participated in the urinary NGAL measurements. PD performed uNGAL measurements and participated in data interpretation. CRP participated in the statistical analysis design and data interpretation. SLG conceived and designed the study, and participated in the data interpretation and manuscript drafting. All authors approved the final manuscript.
